# Family Caregivers of Individuals With Neuromuscular Disease Participating in a Randomized Controlled Trial of a Digital Peer Support Program: Nested Qualitative Study

**DOI:** 10.2196/72141

**Published:** 2025-07-28

**Authors:** Samantha Mekhuri, Craig Dale, Francine Buchanan, Nouma Hammash, Munazzah Ambreen, Sian Saha, Louise Rose, Reshma Amin

**Affiliations:** 1 Department of Paediatrics, Division of Respiratory Medicine, The Hospital for Sick Children, 555 University Avenue, Toronto, ON, M5G 1X8, Canada, 1 6472807374; 2 Lawrence S. Bloomberg Faculty of Nursing, University of Toronto, Toronto, ON, Canada; 3 Tory Trauma Program, Sunnybrook Health Sciences Centre, Toronto, ON, Canada; 4 Patient & Family Engagement, The Hospital for Sick Children, Toronto, ON, Canada; 5 Child Health Evaluative Sciences, SickKids Research Institute, Toronto, ON, Canada; 6 Management and Evaluation, Institute of Health Policy, University of Toronto, Toronto, ON, Canada; 7 Spinal Cord Injury Ontario, Toronto, ON, Canada; 8 King's College London, London, United Kingdom

**Keywords:** family caregivers, neuromuscular disease, peer support, digital, qualitative

## Abstract

**Background:**

Family caregivers have primary responsibility for providing care in the home for people with neuromuscular diseases (NMDs). This may negatively affect caregiver health. Peer support may enhance quality of life and reduce stress among family caregivers, but few trials have been conducted in NMD caregivers. Therefore, we conducted a randomized controlled trial with a nested qualitative evaluation (this report) of a 12-week digital peer support intervention for family caregivers of children and adults with NMD.

**Objective:**

The aim of the study is to gain insights into the perspectives of intervention participants and peer mentors regarding their experiences with the trial’s digital peer support program.

**Methods:**

We conducted a nested exploratory qualitative study (August 2022 to March 2024), recruiting participants who were randomized to the intervention arm of the randomized controlled trial and study mentors. We conducted semistructured interviews via videoconferencing. Homophily theory and the theoretical framework of acceptability informed our analyses.

**Results:**

We interviewed 21 participants and 10 mentors, identifying four themes: (1) program participation motivators, (2) program expectations and appreciation, (3) program appropriateness, and (4) the peer mentor-mentee dyad. We found that participants were motivated to join the program due to existing caregiver burden and social isolation. Participants and mentors appreciated the program’s sense of community and flexible digital format, with participants valuing emotional and informational support. However, challenges in relating to each other’s situations due to participant and mentor heterogeneity in the extent of the care recipient’s needs were perceived to limit benefit.

**Conclusions:**

Peer support was perceived as potentially beneficial in relieving caregiver burden and social isolation, creating a sense of community that provides emotional and informational support. The digital and flexible format was an important facilitator. An important barrier was participant-mentor heterogeneity resulting in reduced perception of homophily. These findings can inform the development of other digital peer support programs to alleviate caregiver burden and isolation and provide emotional relief and informational guidance.

## Introduction

Neuromuscular diseases (NMDs) are a diverse group of rare diseases that affect skeletal muscles, lower motor neurons, neuromuscular junctions, or nerves controlling muscle function [1]. These diseases frequently lead to progressive respiratory failure, requiring mechanical ventilation. Individuals with NMD experience substantial functional impairments, requiring care from family members in the home [2].

Informal family caregivers, including spouses, parents of children, or adults with parents with NMD, provide essential unpaid care and support in the home but risk social isolation and lack of support [3-5]. Providing care and the resultant burden impact both the physical and emotional well-being of family caregivers [6-8]. Peers with caregiving experience can be a crucial source of social and informational support [9]. Homophily theory suggests that when peers share similar experiences, such as caregiving, it can lead to a supportive relationship based on mutual understanding and empathy [10,11]. Support from peers can, in turn, reduce isolation and stress and increase self-efficacy [12-14]. Peer support groups offer information, emotional support, empowerment, sense of community, and improved well-being and coping skills [15,16]. While peer support is often provided in person by community or health care organizations, web-based platforms, such as social media groups, can overcome geographic and time barriers [17,18].

Previous research indicates that caregivers of individuals with NMD can connect deeply with one another, sharing experiences and concerns without needing to explain themselves [19,20]. This connection helps cope with the substantial challenges of NMD while overcoming social isolation [19,21]. Improved coping was demonstrated in a study of caregivers of individuals with amyotrophic lateral sclerosis engaged in digital peer support as part of a palliative rehabilitation program [22]. Caregivers discussed everyday life challenges, found common ground, and learned from shared experiences of loneliness and frustration. Although there are various peer support programs for family caregivers of individuals with NMD in countries around the world, there is a dearth of robust evaluation.

This study builds on our work, in which we feasibility-tested a digital peer support program for caregivers of individuals who were ventilator-assisted, many of whom had NMD [3]. Our aim was to gather insights into the experiences and perspectives of family caregivers and their mentors participating in a randomized controlled trial (RCT) of a digital peer support program [23] to iteratively improve future programs.

## Methods

### Overview

The study was conducted and reported in accordance with the COREQ (Consolidated Criteria for Reporting Qualitative Studies) and Standards for Reporting Qualitative Research: A Synthesis of Recommendations (Checklist 1) [24,25]. We also used the CONSORT (Consolidated Standards of Reporting Trials) checklist when writing our report (Checklist 2) [26]. The 12-week efficacy trial was informed by a pilot feasibility trial [27], and an ongoing RCT of a digital peer support intervention of family caregivers of people with motor neuron disease in the United Kingdom led by LR [28].

### Study Design and Setting

We conducted an exploratory qualitative study of a digital peer support program for caregivers between August 2022 and March 2024. We used semistructured interviews recruiting participants randomized to the intervention arm of an RCT evaluating the 12-week digital peer support program compared to a waitlist control comprising access to intervention materials including discussion session recordings and resources once the 12-week RCT concluded. We also recruited mentors to participate in interviews. The study took place at the Hospital for Sick Children in Toronto, Canada.

### Ethical Considerations

This study received provincial research ethics approval by Clinical Trials Ontario (CTO-3590). Participants and mentors provided written informed consent in accordance with the Declaration of Helsinki before study enrollment. Consent for the qualitative interview participation was obtained when consenting for the RCT. The RCT was registered with ClinicalTrials.gov (NCT05070624). Transcripts were deidentified, and recordings were deleted once transcription was confirmed as accurate. On completion of the study, participants received a US $21.85 gift card in lieu of their time.

### Randomization and Blinding

Participants were randomized 1:1 to the intervention or control arm using an allocation-concealed computer-generated randomization schedule with permuted block lengths of 2 or 4. Study participants, personnel, and outcome assessors were unblinded. A study team member (MA) generated the allocation sequence, and participant enrollment and assignment were made by the research coordinator (SM).

### Sample Size

For the larger RCT, we determined a required sample size of 100 participants (50 per group) based on a minimum clinically important difference of 2.95 on the Pearlin Mastery Scale, an SD of 4.5, 90% power, and 5% α, using assumptions from a previous web-based caregiver intervention study [29].

### Study Participants

Inclusion criteria for participants and mentors participating in the RCT comprised (1) family caregiver of an individual with NMD living in Canada, (2) speaks and reads English, (3) access to the internet and computer or tablet, and (4) mentors were required to complete digital peer support training led by the study team.

We invited all participants in the intervention arm of the RCT and all mentors to participate in this nested qualitative study. Inclusion criteria comprised (1) completion of the 12-week intervention and (2) provided informed consent.

### Digital Peer Support Program

For the trial, participants were assigned a peer mentor with matching based on whether the care recipient was an adult or child with NMD who was, or was not, receiving home ventilation (invasive or noninvasive). Additionally, participants were matched based on the age of the care recipient (0‐5, 5‐10, 10‐18, 18‐40, and 40+ years) and the presence of a similar NMD. No further matching criteria were applied. Mentors were assigned between 1 and 7 participants, with an average of 3‐4 participants per mentor. Multimedia Appendix 1 provides a breakdown of the components of the digital peer support program.

Participants and mentors were instructed to communicate at least once a week through private messaging, calling, or video calling using the aTouchAway app (Aetonix) over 12 weeks. The aTouchAway app was used as a secure means of messaging, calling, or video calling, thereby eliminating the need to share personal phone numbers for communication. This approach was implemented to enhance the privacy of both mentor and participant identity. The aTouchAway app was loaded onto a participant’s or mentor’s smartphone, tablet, or desktop based on their preference.

Mentors were instructed to engage in discussions with participants on a wide range of topics while refraining from offering medical advice. There were no restrictions placed on the topics they could discuss. We did provide some suggestions to mentors as to topics to discuss such as promoting self-care, caring for a loved one, setting boundaries, navigating the health and social care system, managing stress, finding joy in caregiving, and using technology. All study participants and mentors had access to separate group chats, one for participants only and one for mentors only, where they could engage in discussions on various topics, excluding medical advice. Participants and mentors engaged in weekly digital discussion sessions held via Zoom (Zoom Video Communications Inc) moderated by a research coordinator (SM). These sessions addressed various topics including caregiving, navigating the health system, stress management, accessibility for individuals who are disabled, transitioning to adult care, disability and reproductive justice, and mental health. We also had 2 “Ask the Expert” sessions with health care experts (Multimedia Appendix 2). These discussion sessions were recorded to provide participants the opportunity to review the content if they were unable to attend.

### Data Collection

We iteratively developed a semistructured interview guide guided by the theoretical framework of acceptability [30] and homophily theory [31] (see Multimedia Appendix 3 for interview guide). The guide was pilot-tested with the first 2 interviews with minor adjustments to improve performance. All interviews were conducted by 1 author (MA) with a health professional and caregiver background and experience in qualitative interviewing. The interviews were conducted via Zoom. The interviews were digitally recorded and transcribed verbatim. Data collection continued until we reached data saturation, that is, no new themes emerged from the interviews [32].

### Data Analysis

We used directed content analysis [33] starting with a conceptual framework incorporating the constructs of the theoretical framework of acceptability and the homophily theory [30,31]. Following the review of the transcripts, 3 authors (SM, MA, and SS) designed a formative coding matrix based on the predetermined main categories of each theory. Descriptive codes were generated within these categories, and data were reviewed for inductive and deductive themes through constant comparison. To ensure dependability, the coding process was documented and reviewed collaboratively. Confirmability was supported through team-based (RA, LR, and CD) verification of anchor quotes for each theme, promoting consistency and minimizing individual bias. To ensure transparency and reflexivity, we documented our thoughts, decisions, and emotional responses in comments in the transcripts. We explicitly acknowledged how our backgrounds and connection to the topic may have shaped the interpretation of the data through discussion among the research team. MA and SS are family caregivers but remained focused on grounding the findings in participants’ perspectives rather than personal views. In addition, reflexivity was maintained through regular discussions among the research team to minimize potential personal biases in interpretation [34-36]. The thematic framework was revised based on team feedback. NVivo (version 12; QSR International) software was used for coding.

## Results

### Overview

**Figure 1.**
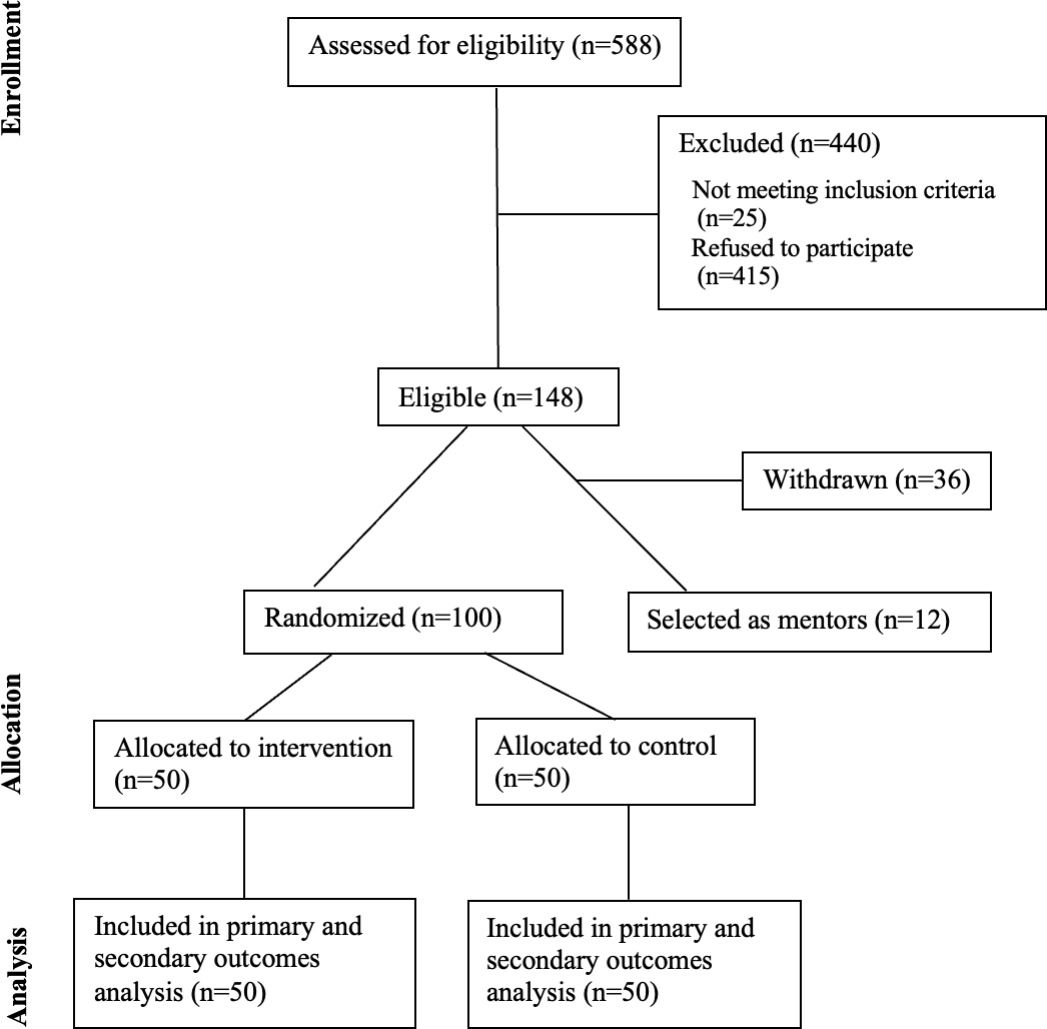
CONSORT (Consolidated Standards of Reporting Trials) diagram for study enrollment and randomization.

We invited all 50 RCT intervention participants after completion of the trial by telephone and email for the interviews; 29 participants declined, citing insufficient time or lack of desire to participate. In total, 21 participants completed interviews. We invited all 12 peer mentors to participate. A total of 1 declined due to lack of time, and 1 died after the program concluded but prior to the interview. We completed 31 interviews, 21 with participants and 10 with mentors, with interviews lasting a mean of 26.0 (SD 10.4) minutes. Figure 1 provides the CONSORT diagram for overall study enrollment and randomization.

A total of 588 individuals were approached for the study; 25 were excluded due to not meeting the inclusion criteria, and 415 declined to participate due to a lack of time or interest. A total of 148 individuals were eligible; 36 participants subsequently withdrew due to no time to participate (n=32) or were no longer a caregiver because of the patients’ death (n=4). A total of 50 peer mentees in the intervention arm, 50 peer mentees in the control arm, and 12 peer mentors were included. Demographic details of participants and peer mentors are presented in Table 1.

**Table 1.** Participant characteristics (N=31).

Characteristics		Participants (n=21)	Mentors (n=10), n (%)
Caregiver age (years), mean (SD)		44.6 (9.8)	43.5 (4.7)
Female sex, n (%)		17 (81)	8 (80)
Caregiver relationship, n (%)			
	Mother	13 (62)	7 (70)
	Spouse	6 (28)	1 (10)
	Father	2 (10)	2 (20)
Caregiving (years), mean (SD)		10.7 (8.4)	10.6 (5.5)
Care recipient, mean (SD)			
	Age (years)	19.4 (19.3)	19.4 (13.4)
	Adult patients	48.0 (17.6)	49.0 (—^a^)
	Pediatric patients	13.0 (6.8)	7.8 (3.3)
Home mechanical ventilation, n (%)			
	Noninvasive ventilation	12 (57)	5 (50)
	Invasive ventilation	2 (10)	4 (40)
	No ventilation	7 (33)	1 (10)
Neuromuscular diagnosis, n (%)			
	MD^b^	8 (38)	1 (10)
	SMA^c^	4 (19)	3 (30)
	Myopathy	3 (14)	2 (20)
	Cerebral palsy	2 (10)	—
	ALS^d^	1 (5)	—
	Other	3 (14)	4 (40)

a Not available.

b MD: muscular dystrophy.

c SMA: spinal muscular atrophy.

d ALS: amyotrophic lateral sclerosis.

Study findings are presented below with four themes: (1) program participation motivators, (2) program expectations and appreciation, (3) program appropriateness, and (4) the peer mentor-mentee dyad. See Table 2 for theme definitions and Multimedia Appendix 4 for supporting quotes. The dimensions of the theoretical framework of acceptability reflected in the themes include affective attitude (participants’ feelings about the intervention), burden (perceived effort required to participate), intervention coherence (understanding of the intervention and how it works), perceived effectiveness (belief in the intervention’s ability to achieve its purpose), and self-efficacy (confidence in participants’ ability to perform the required behaviors) [30].

**Table 2.** Qualitative framework.

Theme	Definition
1. Program participation motivators	Motivation to join the peer support program due to the absence of social and emotional support in their role as the primary caregiver.
2. Program expectations and appreciation	Pre-existing expectations and expressed appreciation for the program because of filling gaps in social connectivity.
3. Program appropriateness	Infrastructure, design, and operationalization of the digital peer support program.
4. The peer mentor-mentee dyad	Matching, communication, and connections.

### Theme 1: Program Participation Motivators

Participants and mentors highlighted significant caregiver emotional stress caused by the lack of social and emotional support faced daily as a primary caregiver, motivating many to join the peer support RCT. Participants cared for individuals with various types and severity of NMD, ranging from advanced, complex cases to those in early stages. Many participants were mothers caring for more than 1 child with other children who did not have NMD. Participants reported significant burden and stress related to their care recipient’s diagnosis and level of dependency. Some participants and mentors described being able to secure a support system to address stress from family and friends, while others described the absence of a social support network.

*I don’t know, it’s very complex how you do it, but some days, you feel like you float and try to stay alive and then other days you’re doing good. So each day at a time. Sometimes each hour at a time.*

[Participant 6]

Several participants described joining other support groups previously, especially web-based groups. Many reported involvements with an NMD-related charity with a few participants and mentors being in a leadership role for these organizations’ support groups.

*I have a great support system. My husband, we’ve been together since we were 17. ... we have teenagers, and they’re usually a really good help as well. We have some families that are in the medical field as nurses, so they’re great resources—we have a really good network of support people for us*.

[Participant 7]

Other participants described not having any type of support and were motivated to join our trial to expand their caregiver network, thus relieving social isolation.

*Well, I don’t really have family support—I’m on my own, it’s not easy. I’m always looking for extra caregiver support programs, where to connect with people that are like me—because out there in the real world, nobody really validates, supports, or understands.*

[Participant 7]

### Theme 2: Program Expectations and Appreciation

#### Program Expectations

Participants and mentors shared their pre-existing expectations of learning new things and connecting with others in similar situations. Some participants described not having any expectations and joined with an open mind. Mentors had an expectation of passing on knowledge and giving participants emotional support without providing medical advice.

Some participants felt that they had expectations that were not met. One participant perceived their mentor as a peer with similar needs, rather than someone who could provide advice and guidance, and felt that their experience would have been different with that mindset from the start.

Another participant felt that the program was not relevant to their caregiving situation and that they did not need personalized support. A few misunderstood the program’s purpose, thinking it was purely about gaining informational resources rather than emotional support.

*I think when I kept hearing the word mentor I went into it thinking I’m going to have somebody that might be able to guide me a little bit—or somebody that I can lean on and say, “Hey when do you have your grants for this?,” but being put with somebody that I found was more of a peer, because I didn’t go into it with that mindset, it didn’t jive with me.*

[Participant 6]

#### Program Appreciation

Participants expressed an appreciation for the digital peer support program due to gaps in their social networks and lack of peer support, contributing to positive attitudes toward the intervention.

*I think this is a really good [program] to help us to understand what’s going on. So being connected with other people that are going through the same thing is really helpful.*

[Participant 8]

Most participants perceived real value in the peer support program for fostering personal connections and filling a gap not met by family and other social media groups.

*What I got out of it was the ability to meet people that have similar shared experiences and a place that if I need to go and talk to somebody—and an opportunity to learn things about how to better cope or manage my wife’s condition.*

[Participant 16]

Some participants shared that they accrued more knowledge than they thought they would. When asked if they would continue with the program if it was extended, these participants said yes and would recommend it to a friend. Mentors also noted a bidirectional learning relationship that resulted in their positive attitude toward the program.

*Quite a good program, emotionally helpful as well. I really enjoyed going through this program. It’s a first for me for sure—you learn from your mentee [participant], you learn from the different type of workshops or seminars that we had—then at the same time, you had support from the coordinators as well*.

[Mentor 8]

Although participants had limited time to participate, they adjusted their schedules to accommodate the peer support program, given its perceived value.

*I just put aside the time, which I don’t have a lot of time, that’s for sure. Because like I said, I have no other help. But I think this is so important, I almost treated it like self-care. I don’t do self-care enough for myself, I have no time as the sandwich caregiver, but I really put the time aside and told myself, you know what this is for me and I’m going to make it happen.*

[Participant 7]

### Theme 3: Program Appropriateness

Several participants appreciated the digital aspect of the program and the flexibility to connect with their mentor from anywhere at any time. Many participants valued information from the weekly discussion sessions and that the sessions were recorded and accessible at any time.

*I did find it valuable, and you can make connections and talk to people virtually—I think our world has become this virtual world. So, it’s becoming more normal. It’s just that this makes it easier to juggle. I think if it was more in person, then it might be more difficult to go to.*

[Participant 4]

Some participants perceived program components more negatively, citing issues with the app’s incompatibility with Android phones in terms of receiving message notifications and glitches with text messages disappearing when typing, resulting in retyping messages.

*I have an Android phone—notifications don’t exist. So, you have to go into the app every time just to see if somebody’s notified you.*

[Participant 10]

Conversations between participants and mentors were purposefully self-directed in the program. However, this was dependent upon the participant and mentor to initiate and maintain a minimum of once-a-week interaction. One mentor felt that more time to build a relationship with their mentee on program commencement would have been helpful to develop a meaningful relationship. One mentor felt that the program seemed like a forced interaction (ie, “a scheduled conversation”) rather than spontaneous, open communication.

*I guess it feels more like a forced interaction with this program here versus a larger Facebook group where people can ask what they feel like asking when they feel like asking. It can be anonymous; it can be however way you want to kind of connect. Whereas in this case, I think it was a bit more scheduled to have a conversation.*

[Mentor 4]

Participants and mentors shared various perspectives regarding the structural aspects of the peer support program’s intervention. Participants provided diverse feedback with regard to the weekly discussion sessions. Some shared that they had hoped for additional scheduling options for the weekly group discussion sessions rather than held on a fixed day and time. These participants mentioned having other commitments or having to provide care for their care recipient, which precluded their attendance.

Many participants appreciated the discussion session content with the topic variety and guest speakers. However, participants and mentors expressed challenges with combining pediatric and adult family caregivers in the weekly discussion sessions, given experiential differences. Some participants perceived some sessions as difficult to engage in, lacking utility, or being unrelatable to their own situation.

*I just found that was not successful at all having pediatric and adult caregivers present. Because it’s two completely different worlds that you’re dealing with.*

[Mentor 10]

Despite these concerns, for the most part, discussion forums were perceived as enjoyable and inspirational.

*There was one speaker, her son is a quadriplegic and oh my god not only did she give this fantastic presentation, which I was like when did she have time to pull this together—her story was so humbling. You couldn’t help but walk away and be like how is this woman living life, you definitely go like “if she can live life, I can live life*.”

[Participant 10]

When asked for recommendations to improve the program, participants suggested the following: (1) separating adult and pediatric family caregivers into respective discussion sessions, (2) breakout rooms encouraging greater participation and engagement, (3) sessions moderated by a family caregiver with lived experience, and (4) improved moderation to avoid individuals dominating the conversation. Participants and mentors gave suggestions for additional weekly discussion session topics including workplace supports for patients, puberty, intimacy, personal support workers and home nurses, financial support and resource navigation, transitioning to adulthood, advocating, and addressing medical equipment questions.

### Theme 4: The Peer Mentor-Mentee Dyad

Participants and mentors shared experiences with regard to mentor-mentee matching reflective of homophily theory, communication, and connections. While most participants felt supported by their mentor, many reported difficulties in establishing personal connections, citing time constraints and differing levels of dependency of their care recipient as key obstacles. Many participants perceived suboptimal matching and feelings of incompatibility with their peer mentor. Suboptimal matching arose from age differences between mentors and participants and participants having comparable levels of caregiving experience to their mentors. This led to not perceiving support or even feeling they were supporting their mentor in certain situations. Many participants wanted a mirrored relationship with their mentor, which they defined as the same care recipient disease, acuity of care needs, and similar levels of dependency. This was described as the most important factor regarding participant-mentor compatibility with resultant program engagement.

*I think if I was matched with somebody that understood me a little bit better, or was in a similar type of situation, then I would have made more of an effort to be part of that program, and I think there would be usefulness in that.*

[Participant 12]

Some participants conveyed challenges with making time to communicate, as mentors were not always as available as expected. Both participants and mentors expressed feelings of guilt when reaching out, as they were aware of the caregiver burden the other was facing and not wanting to add to the challenges already being dealt with.

*I was more worried that it was putting an undue burden on them.*

[Mentor 3]

With respect to communication between the mentor-mentee dyad, various topics were discussed such as experiences with doctors, navigating the adult world for pediatrics, questions about funding, agencies, stress relief, advocacy, and life in general.

*Some of the children had tracheostomies, some had different types of disabilities. What got me connected is the parents is just them speaking about their day, and sometimes how stressful it could be, or sometimes how happy it can be and actually relating to them. That’s what got me connected to some parents.*

[Mentor 1]

Nevertheless, developing a fulfilling relationship was difficult when conversations were perceived as surface level only with minimal effort used to get to know the other person. Additionally, mentors handling numerous participants reported challenges in connecting with all of them. Despite these perceived imbalances in the mentee-mentor dyad, most participants valued the program for the support and sense of community it provided.

In terms of the connection between participants and mentors, mentors offered support in various ways.

*I think that the mentor mentee relationship is very important. That’s where people feel validated support. I just think it’s so important. I’m stressed and anxious on a good day, most of the time being honest. So having a program like this is very beneficial*.

[Participant 7]

Some mentors highlighted being able to assist participants by offering information about community services and advice to cope with stressful situations.

*One example was I had one of the mentees struggling with funding a particular equipment. I had to share some funding agencies that we got help from.*

[Mentor 8]

One participant felt that regardless of differences with their peer mentor, there was still some common ground to connect. Some participants also felt connected with other participants in the discussion sessions and could support others based on the mere fact of being a family caregiver and dealing with similar struggles in their daily lives.

*It was very interesting and informative. My biggest positive takeaway was knowing that there are so many more of us out there. We are an unrecognized community—so it was easy for us to appreciate what all the other caregivers were doing—we’re all comfortable enough with our own burdens.*

[Participant 17]

## Discussion

### Principal Findings

This qualitative study nested within an RCT explored the experiences of participants and peer mentors caring for an individual with NMD and participating in a 12-week digital peer support program. We identified four themes: (1) program participation motivators, (2) program expectations and appreciation, (3) program appropriateness, and (4) the peer mentor-mentee dyad. Caregiver burden and lack of social support motivated participants to join the peer support program. Participants and mentors had varied expectations including the desire to learn new information, connect with others in similar situations, and receive emotional support. Some participants felt that their expectations were unmet, specifically regarding the mentor-mentee dyad, with a few participants misunderstanding the program’s focus on emotional support rather than solely informational guidance. Participants and mentors generally appreciated the program and support provided, expressing a preference for continued participation should the opportunity arise. While participants appreciated support from mentors and the digital format due to logistical constraints, challenges to acceptability included the heterogeneity of the participant group, varying severity of NMD, time limitations, and lack of structured engagement in discussion sessions. Additionally, some participants experienced difficulties in establishing meaningful connections, often due to differences in caregiving experience, age, or the level of dependency of their care recipients.

Similar to recent research among family caregivers of individuals using mechanical ventilation, participants shared feelings of burnout and stress associated with being a caregiver of an individual with NMD [37], which motivated them to participate in the RCT. While some participants and mentors had prior experience with peer support, for others, this program was a novel experience. Several participants valued this structured peer support program, as very few programs exist outside of internet-based support groups. Existing research has found that peer support can alleviate feelings of isolation, provide emotional relief, reduce anxiety, enhance well-being, and offer valuable social support [3,38-41]. Although most participants felt supported by their mentors, some participants did not perceive benefits from the program, citing difficulties in “opening up” to their mentors, a lack of perceived need for support, or feelings of some incompatibility with their mentors. This suggests that responses to and the need for peer support are somewhat heterogeneous and dependent on the mentor-mentee relationship.

In our study, we found homophily [31] (defined as the condition whereby similar people are attracted to one another at a higher rate) to be a key mechanism of intervention acceptability. Therein, a mismatch between mentor-participant characteristics in relation to the disease severity and disease type of the care recipient functioned as a key barrier. Participants were matched to peer mentors based on whether the care recipient was an adult or child, age of the care recipient, similar NMD diagnosis, and use or nonuse of home mechanical ventilation. Other studies on peer support for care recipients have considered their preferences for matching. For example, Pistrang et al [38] incorporated care recipients’ preferences, including their preferred newspaper as indicators of their “world view.” In this study, they also met with participants to get a sense of who they were as an individual and their support needs. Implementing such in-depth matching could potentially mitigate the lack of homophily, but such matching is challenging to achieve with the availability of a reasonably small pool of mentors, given that many of our participants were caregivers of care recipients with rare diseases.

To date, the acceptability of delivering digital peer support has been underexplored in vulnerable, medically complex populations, including those with NMD. We found that the acceptability of the digital format was facilitated by its flexibility, especially through providing access to discussion session recordings. This was particularly appreciated by participants with employment outside the home or substantial caregiving responsibilities, as it allowed participation based on their schedules, which has been shown in previous studies [15,42-44]. However, some participants found the fixed scheduling of the discussion sessions to be somewhat inflexible, with a preference for additional scheduling options. However, given resources around moderator availability, we were unable to offer such options. Future research should explore how to balance the flexibility of digital formats with the interpersonal connection and trust-building that in-person meetings may provide.

The inclusion of participants caring for adult and pediatric care recipients in the same discussion group in our study was perceived negatively. This has also been highlighted in other research with adult caregivers focusing on feelings of grief and loss, whereas pediatric caregivers focus on their child’s developmental pathway [42]. Therefore, increased heterogeneity in discussion groups may negatively influence participant outcomes [42]. Future research should explore homogeneity within peer support groups to enhance their effectiveness and relevance for participants.

### Strengths and Limitations

Strengths of this study include the inclusion of family caregivers representing a range of NMDs and the use of homophily theory and the theoretical framework of acceptability to inform our analyses. Concepts derived from these theories helped to sensitize the research team to relevant issues, processes, and interpretations that might otherwise not have been identified. The interviewer’s positionality as a family caregiver and a health care professional is also a strength.

Our study has limitations. First, recruiting a heterogeneous sample of caregivers for both children and adult NMD care recipients reduced the sense of homophily, which in turn affected perceived acceptability. Second, fewer than half of the participants agreed to be interviewed, with those who declined citing lack of interest or being too busy. As a result, our findings may be biased, as we did not capture the perspectives of participants who may have experienced less benefit from the program.

### Conclusions

In summary, our nested qualitative study provides valuable insights into the experiences of mentees and peer mentors caring for individuals with NMD in a 12-week digital peer support program. The study highlights the significant emotional stress faced by caregivers of individuals with NMD and demonstrates the potential of a peer support program to alleviate caregiver stress. Caregivers were motivated to participate in the program due to expectations of gaining new information and connecting with others in similar situations. While participants generally valued the support provided by mentors and appreciated the digital format due to geographic and temporal constraints, several challenges to acceptability emerged. These included participant heterogeneity and challenges with mentor and participant matching, resulting in difficulties in forming meaningful connections. Refining the structure and participant matching in future studies may enhance the effectiveness of peer support for family caregivers.

## Supplementary material

10.2196/72141 Multimedia Appendix 1 Overview of the digital peer support program.

10.2196/72141 Multimedia Appendix 2 Weekly discussion topics.

10.2196/72141 Multimedia Appendix 3 Semistructured interview guide for peer mentors and caregiver participants.

10.2196/72141 Multimedia Appendix 4 Supporting quotations.

10.2196/72141 Checklist 1 COREQ (Consolidated Criteria for Reporting Qualitative Research) checklist.

10.2196/72141 Checklist 2 CONSORT (Consolidated Standards of Reporting Trials) checklist.

## Abbreviations

CONSORT: Consolidated Standards of Reporting Trials
COREQ: Consolidated Criteria for Reporting Qualitative Studies
NMD: neuromuscular disease
RCT: randomized controlled trial
