# Supplementary material for: Family Caregivers of Individuals With Neuromuscular Disease Participating in a Randomized Controlled Trial of a Digital Peer Support Program: Nested Qualitative Study
Source: J Med Internet Res. 2025 Jul 28;27:e72141. doi: 10.2196/72141 (PMC12303555; doi:10.2196/72141)
Supplement: Multimedia Appendix 1 [file jmir-v27-e72141-s001.docx]

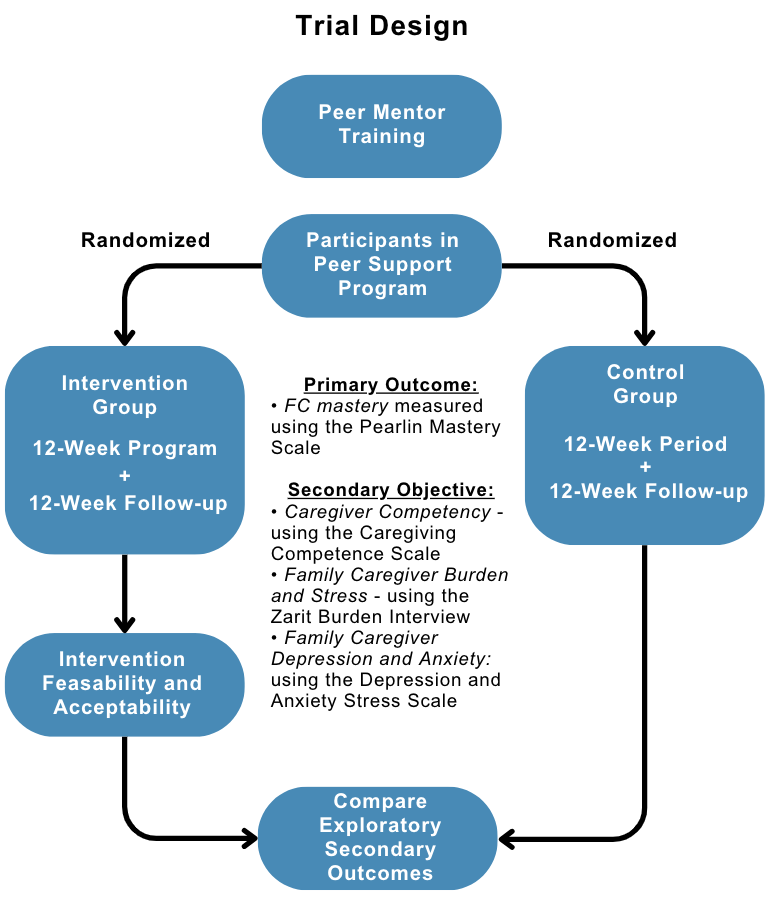


| **Intervention Feature** | **Details** | **Peer Mentor Frequency** | **Participant Frequency** |
| --- | --- | --- | --- |
| Informational Resources | PDF/weblinks/videos available on app | Accessed as needed | Accessed as needed |
| Discussion Forum | Enables asynchronous peer-to-peer contact | ≥ 2 times/week | ≥ 2 times/week |
| Weekly Live 1-hour Forum | Zoom sessions to discuss relevant topics  -Moderated by research team | Weekly | Weekly |
| Private messaging | Peer to peer messaging via the aTouchAway™ app | Weekly contact  -Encouraged to respond to messages within 48 hours  -Can interact with 1 or several participants | ≥ 1/week to mentor |


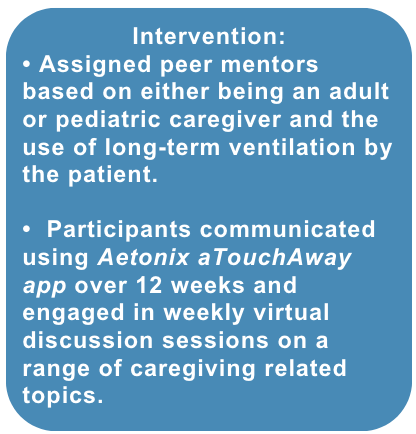


Abbreviations: FC, family caregiver.

**Components of the Intervention**
