# Supplementary material for: Family Caregivers of Individuals With Neuromuscular Disease Participating in a Randomized Controlled Trial of a Digital Peer Support Program: Nested Qualitative Study
Source: J Med Internet Res. 2025 Jul 28;27:e72141. doi: 10.2196/72141 (PMC12303555; doi:10.2196/72141)
Supplement: Multimedia Appendix 2 [file jmir-v27-e72141-s002.docx]

- Introduction to Peer Support
- Promoting self-care and how you look after yourself as a carer
- Caring for your loved one
- Setting boundaries as a caregiver
- Navigating the health and social care system
- Caregiving and other relationships
- **Ask the Expert - Speaker:** Muscular Dystrophy Canada
- Managing stress
- Social Activities
- Finding joy in caregiving
- Navigating technology
- **Speaker - Caregiver and Teacher, CanChild Centre for Childhood Disability Research​:** Stress Management and Self-Care/Advocate for Patient Rights and How to Speak to Healthcare Professionals​ and Communication with Clinicians, with our children, and with ourselves
- **Speaker - Research Patient & Family Engagement Coordinator:** Mantras, Models, and Mnenomics – Caregiver Toolbox​
- **Speaker - Social Worker:** Caregiving from the Patient Perspective, Being Successful as an Individual with NMD​
- **Speaker - Canadian Neuromuscular Disease Registry:** What is CNDR and How to Get Involved​
- **Speaker - Manager of Independent Living Resources​, Independent Living Waterloo Region:** Accessibility
- **Speaker - Neurologist:** Transitioning to Adult Care​
- **Speaker - Professor at Toronto Metropolitan University in Disability Studies:** Disability and Reproductive Justice ​
- **Speaker - Psychotherapist and Spiritual Care Provider:** Mental Health Management​ and Mindfulness
- Experience with the Peer Support Program
