# Supplementary material for: Family Caregivers of Individuals With Neuromuscular Disease Participating in a Randomized Controlled Trial of a Digital Peer Support Program: Nested Qualitative Study
Source: J Med Internet Res. 2025 Jul 28;27:e72141. doi: 10.2196/72141 (PMC12303555; doi:10.2196/72141)
Supplement: Multimedia Appendix 3 [file jmir-v27-e72141-s003.docx]

**Connecting Caregivers: A Randomized Controlled Trial to Evaluate Virtual Peer-Support for Family Caregivers of Individuals with Neuromuscular Disease (Mentees)**

[Introduce study, interview’s purpose, their consent to be interviewed and arrangement of a convenient time to call; all prior to interview either by phone or e-mail]

*Thank you for taking the time to speak with us today. I am going to ask you about your experience with the virtual peer-support program. Your valued feedback will help us to better understand how to best provide a virtual peer support to family caregivers of children or adults. The interview is expected to take approximately 30 to 60 minutes and your answers are confidential.*

*Background of Mentee*

1. Can you tell me about yourself?
2. Can you tell me a bit about the support system you currently have (i.e., social media)?
   1. Are you a part of any other peer support groups?

*Experience with Program*

1. Can you share with me your experience with the virtual peer support program?
   1. How did you like the program?
   2. What were your expectations and were they met?
   3. How did the program fit into your day/week?
2. Can you tell me about the communication between you and your mentor?
   1. How often did you communicate with them?
   2. What were the dynamics of the relationship?
3. What kind of issues did you discuss with mentor and did you feel supported?
4. Can we talk about a bad day you may have had since you joined the peer support program? Maybe where the family member you are caring for had a medical emergency or just a day that went sideways.
   1. Did you connect with your mentor on this day? Do you think the virtual peer support program could have worked better for you on that day?
5. Was there a time where you had to support your mentor with the issues they may be dealing with?
6. How were the discussion sessions?
   1. Did the topics cover everything?
   2. Are there any topics you wish could have been added?
   3. Is there anything you would change about the sessions?
   4. Would you prefer if this was in person?

*Suggestions for the Program*

1. If the virtual peer support program was being fully changed, what are one or two things you would keep the same? What would you change? Would you want to continue? Would you recommend this to a friend?
2. We are coming to the end of the session, is there anything else you would like us to know, that you haven’t had a chance to share?
