# Supplementary material for: Family Caregivers of Individuals With Neuromuscular Disease Participating in a Randomized Controlled Trial of a Digital Peer Support Program: Nested Qualitative Study
Source: J Med Internet Res. 2025 Jul 28;27:e72141. doi: 10.2196/72141 (PMC12303555; doi:10.2196/72141)
Supplement: Multimedia Appendix 4 [file jmir-v27-e72141-s004.docx]

**Appendix 4:** Quotations

| **Themes** | **Quotations** |
| --- | --- |
| 1. Program Participation Motivators | Caregiver loneliness, loss of job, caregiving impact  “We have accepted the fact that we are facing this issue—and the kind of environment has stabilized for us in terms of support, in terms of emotional issues and the way we handle day in day out with our son, we have changed our house twice because of that. But overall, we are able to provide him the quality of life, which he needs and he feels satisfied and okay with everything. - Participant-5”  Access to any support  “During the first few years after his diagnosis, I, myself, even working in the pharmacy industry, I've never heard of Pompe. It was an entirely new world to me. I did as much research as I could, but back then there was not enough information. I had no support system. I had to educate my family. But they had their own lives. They weren't able to help, they helped as much as they could, financially. But, otherwise, they weren't able to because we didn't understand what Pompe really was about and how to deal with it. – Participant-5”  Experience with other peer support groups  “I had an organization and I was running a lot of peer support groups, and we were getting families together. So I know a lot of Duchenne families, I have a good network of friends. I also have another Duchenne mom whose kids the same age as O, and she lives in the area. So we have a really great friendship and she's really great as a mentor, not a mentor, but we support one another more--I'm actually an advisor for many of the organizations and I'm part of the World Duchenne organization. So I actually know quite a lot of families internationally that we get together and we talk but we also provide advice to pharmaceutical companies on how to run their trials and, the drug development process--I was on Facebook, I was connected to all the Facebook groups, but I actually needed to take some time and just to not see any of it for a while. – Participant-12”  “There's a couple of Facebook groups that I'm a part of that are specifically myotonic dystrophy, where we have a place to vent or ask questions and seek advice or at least opinions--then I have a couple of good friends that are in the same boat, not quite myotonic dystrophy but muscular dystrophy or multiple sclerosis. So similar that we deal with, talk, complain, and bounce ideas off.” - Participant-11 |
| 2. Program Expectations and Appreciation | Pre-program expectations  “I also wanted to join this group so that I could just hear other people who are going through something similar, obviously, people run the gambit of who they're taking care of. But I wanted to hear and learn from other people, here's some good speakers, and if I came away with a few nuggets of information that could help us in the future, I will be satisfied, and that's what I feel I did. – Participant-10”  Overall experience with the Peer Support program  “I'll preview this by saying I like the peer support program. I like the fact that I could talk to other people that have these issues, I learned stuff from them, someone may have, "Hey, I tried such and such drug and it helped", or, you know, "I bought a different mask for the CPAP machine, and it's much more comfortable". That kind of knowledge is beneficial--Again, I don't know if you've looked at my survey results, but in my opinion, I don't see that they changed much from beginning to end. But that does not mean that this was not a beneficial program. – Participant-16”  Value or meaning of access to peer support  “I think the one thing for a lot of caregivers that I've found is that we all feel isolated, and we're looking for connections. So even trying to make groups available where you can connect with these people maybe outside of these groups, or even in person as a regular or monthly thing so that you could meet with people in person. – Participant-4” |
| 3. Program Appropriateness | Peer support cherished liked-components  “I thought all of the speakers were really good, worthwhile speakers, really interesting--some of them kind of hit home more than others. I really loved to be honest the caretakers themselves talking about different...it's almost like homemade presentations, but they were so good. They had thought about these things so well, it's those ones that seems to really hit me hard, or hardest, or connect with me--I was so appreciative, almost feeling I wasn't alone. – Participant-15”  Components not useful  “One thing I will say- I have been involved with support groups in the past and the level of emotional connection and level of trust is considerably better face to face. Zoom doesn't cut it--. I'm in my 60s, I'm old school, right? Maybe for a millennial, or someone in their 20s, they can derive an emotional connection through a Zoom meeting. Doesn't work that way for me. – Participant-16”  Program recommendations  “I would say that I love the idea of having a family mentor. But I think a little bit more thought has to go in on how you're pairing up families, and how you're finding a mentor for somebody. So ideally, if you can find individuals with the same condition, and around the same kind of stage, it's almost you need to interview the people who are applying for the program, it's almost like a dating service, right, you're doing a match making service. To pair up like-minded people and it may or may not work too because personalities are different, and some people have a natural ability to get along, others might be a little bit more difficult. So there's always a trial period. – Participant-12”  How timing worked for the program  “I don't know if I could commit again to doing a full another three months or so just because I usually have a lot going on. I feel I've taken from it and felt a lot better after taking the program. But I think this three month thing was a pretty good time period for doing the support group. – Participant-20”  “It required a little bit of manipulation on my schedule and I mean that was okay. Again, because I feel very strongly about participating. I was more than prepared to move some things around and perhaps say no to some other things. Because this was important and it still is important.” – Participant-17  How often the sessions were attended  “I didn't get to tune into all of the discussions, I did miss I think two or three of them, but the ones that I did catch were great, and I loved them. – Mentor-7”  Quality of sessions  “It was fun at the beginning. We were all shy. Once we got less shy, the discussions seemed more discussion-like, as opposed to one person answering question, and then another person answering the question. We were talking to each other, which was pleasant as well. – Participant-17”  Session Recommendations  “I would do probably breakout rooms. Just to get everybody participating, as opposed to you have the speaker and then are there any questions? Because sometimes not everybody wants to participate. I think a breakout room would be good --because you would be more inclined to open up if you're in a smaller group, if there's 10 to 15 online, then you could get three or five people or three groups, to participate and then you come back and then everybody gives their feedback as to whatever was discussed and how they felt, and I feel that would get more participation. – Mentor-5”  App usability, interactions, quality, and recommendations  “The app is really easy to maneuver. I really like it. I like it when you're getting a message and dings on my phone. I could just automatically sign in, and go through all of the people that I'm speaking to – Mentor-1” |
| 4. The Peer Mentor-Mentee Dyad | Mentor-mentee matching and relationship  “My mentor introduced herself and she seems so nice--she said I think it was her daughter that she cares for one of them. She just had so much going on her life and her daughter needed a lot more support, like trach or something like that--I know this is awful. But she was so willing to help but I just felt oh my gosh, she needs to give her kids so much more than I even need to give. So I just felt that how could I be asking her for help? Then I also felt the situations were a little bit different. So I didn't use that side of it and I thought I would. - Participant-15”  “[My mentor] was a parent of a child with special needs, and it was the same situation as me, but her child is much younger. So, I feel I've already passed some of her experiences. I probably have more to offer her than she has to offer me.” – Participant-4  Connections made or not  “Maybe that’s the problem, she has a five year old that's just doing better than mine. She's asked me things about, does he have trouble, or when you have trouble with fitness or something, and for me, my child will never get to that point, because he just had more abilities than my child does. So I think maybe that was more difficult, too, because here's our kids and we're at the same stage, and we're at the same level, and yet her kids doing so much more than mine. So maybe that was part of the difficulty in my end, was just it's different when you talk about a kid who's able to communicate and move around or sit up or do things, and there's my child that... he can't sit unassisted, and he can't, he's nonverbal. He's not expressing he doesn't even smile. So maybe it's just the level of complexity that I have. That just made it so hard, because I don't think unless you have a child that's medically fragile, it can be hard to understand what somebody's going through. - Participant-6”  “My mentor, I felt right from the beginning, he had a genuine interest in who I was and what my situation was. He was very understanding, kind, and caring, and had a great sense of humor and just made me feel super comfortable and right away I felt very comfortable to open up and to start discussing about my life and about things that I struggle with, and the things that I've gone through with my son and some of the difficulties he faces. – Participant-13”  Similarities-dissimilarities in matching  “But even though it was a neuromuscular disorder, I couldn't really relate to her because her questions were more direct to her son's condition and I had no knowledge of that condition. So I couldn't even assist her with that. Because she was asking questions relating to that, and I'm like, well, my son doesn't have that condition, I don't know. So I found that very challenging. – Mentor-5”  “The people I was paired up with at the beginning, we did have similar things whether they were a caregiver for a spouse, as opposed to a parent or child type. So I did find that was convenient. Their locations which they ended up divulging to me, I don't know if that was included in the pairing, but it was good because of the size of city and proximity. I was somewhat familiar with some of the services for one and then I was familiar with, you know, large city access for the other one, if that makes sense. I don't know if that was intentional or just coincidence, but I felt that was a good fit. – Mentor-10”  Relatability, authenticity, supporting human connections  “I try not to be overly positive because there's lots of challenges in my day-to-day life. But sometimes when things are going badly for people, it's hard for them to hear if things are going well for somebody else in a similar scenario. So, I found that hard to balance. – Mentor-3”  Homophily within the groups  “We have so many things similar going on with the people with the same disorder, degenerative, what they're going through. It has been quite helpful--this group brings one-on-one, a person in front of you to discuss what they're going through. We have the similarities, and you're not alone in the boat. – Participant-3”  “I did meet a woman who reached out to me who had a nine month old also with SMA and also had Zolgensma and now we are friends, we're friends and we've never met. This study actually allowed me to make a friend who understands what I'm going through which we both talked about, it was really nice. I would have never found that if it had not been for this group-- she's younger than me but we both bonded over the fact our babies are six-seven days old and we got a call that changed your whole life and, no one would like to have a child and then find out that they're sick and feel responsible for that. – Participant-10”  Mentor-mentee relationship dynamics  “I had a mentor and she was a very interesting, very kind, a helpful person, and she made suggestions. But I guess part of the concern I would have is based on how the study was set up- that mentor was mentoring a whole bunch of people. That mentor also has a person with challenges that she has, she is also a caregiver. If I was to put my mentor hat on, I would suggest that perhaps this mentor did not have sufficient time to fully devote herself to the mentoring process. Again, I don't have a problem with that because, it's a study, the mentor has her own life, the mentor has a partner who has a neuromuscular condition as well. So, I mean, all of these things are very important to her, I'm fine that I take second place with that. – Participant-16”  “He really supported me. We exchanged good information. He sent me valuable information about other organizations that can support extra physiotherapy and kind of things for our son. So I think it was really good conversation and it was really good being connected with him. - Participant-8”  Examples of peer support to resolve needs and challenges  “I believe she was quite anxious about her son needing a device that--she hasn't been exposed to. It was a big concern, because he had to do imaging before they could recommend this device because it could be a blockage or something of the airway. She reached out and was very anxious, and didn't know what to expect. That piece of it I could support her because my son is on that device, and I had to go through the process. So I felt she received that support, because the response went back and forth. I did check in with her after her son did receive the device and she was working through it. I felt both ways, I was able to support her and she did receive it, because of her feedback. – Mentor-5”  How well the program functioned in gaining support  “But I wouldn't say it really alleviates the challenges I have faced or my level of sadness around the situation because, although that information is useful, none of it actually solves the problem, and the things that I need, I would want to see happen to help improve her quality of life are outside the realm of a support group. It is not something a support group can provide-- I would say that any support group was better than no support group. While the causes of my concerns were not alleviated, I mean, in terms of depression, I'm going to say, really not much has changed. – Participant-16”    “Because, the trial is only for not that long, the time period is not that long, right? So I think long term, it may be okay--it may work, cause in the long term people maybe they have time, then they will reach out. But we are only talking about few months now. Maybe it didn't show the full picture of how useful it will be, right? – Mentor-6” |
